# Determinants of Plasma HDL-Cholesterol Concentrations in Prepubertal Children: Insights from Two Decades of Research in a Spanish Pediatric Cohort

**DOI:** 10.3390/ijms27219984

**Published:** 2026-11-07

**Authors:** Olga Pomares, Henar Ortega-Senovilla, Leandro Soriano-Guillén, Carmen Garcés

**Affiliations:** 1 Lipid Research Laboratory, Instituto de Investigación Sanitaria Fundación Jiménez Díaz (IIS-FJD, UAM), 28040 Madrid, Spain; olga.pomares@iis-fjd.es; 2 Department of Chemistry and Biochemistry, Facultad de Farmacia, Universidad San Pablo-CEU, CEU Universities, 28660 Madrid, Spain; henar@ceu.es; 3 Department of Pediatrics, Instituto de Investigación Sanitaria Fundación Jiménez Díaz (IIS-FJD, UAM), 28040 Madrid, Spain; lsoriano@fjd.es

**Keywords:** *CETP*, gene–diet interaction, genetic variants, HDL cholesterol, leptin, obesity, prepubertal children, sex differences

## Abstract

High-density lipoprotein cholesterol (HDL-C) is a well-established cardiovascular risk marker; however, its determinants in childhood remain incompletely understood. The prepubertal period provides a useful window for studying the determinants of HDL-C because it is free of confounders present in other age groups. In this review, we summarize the findings on the plasma HDL-C determinants in Spanish prepubertal children aged 6–8 years from decades of research in the Four Provinces Study. Children with obesity had lower HDL-C concentrations, but BMI was only weakly associated with HDL-C, whereas markers of adipose tissue function, particularly leptin, showed stronger and potential sex-dependent associations. Among common variants in genes directly involved in HDL metabolism, *CETP* TaqIB emerged as the main genetic predictor, although it explained less than 5% of HDL-C variability. Variants in genes related to adipose-tissue biology (*LEP*, *PLIN1*, *PLIN2* and *DLK1*) were also associated with HDL-C in a sex-specific manner, and some associations were modulated by circulating leptin. Dietary intake made only a modest contribution to HDL-C variability, yet fat intake modified the effect of the *LIPC* −514C>T variant on HDL-C. Overall, these findings support an integrated adipose tissue–HDL axis in childhood and suggest that sex may influence this relationship even before puberty.

## 1. Introduction: HDL-Cholesterol in Children and Low Rate of CHD in Spain

Atherosclerotic lesions begin to develop in childhood [1,2], and the factors that influence their progression have therefore attracted considerable attention. Plasma high-density lipoprotein cholesterol (HDL-C) concentrations are inversely associated with the development and progression of atherosclerosis, and childhood lipid levels, including HDL-C, predict cardiovascular events in adulthood [3,4,5,6]. Large epidemiological studies in adults have consistently shown an inverse association between HDL-C and the risk of coronary heart disease (CHD) [7,8]. However, Mendelian randomization studies and the neutral results of HDL-C-raising trials have questioned a direct causal role of HDL-C, and extremely high concentrations have even been associated with increased mortality [9,10]; current research therefore focuses also on HDL functionality [4,5]. At the population level, the higher HDL-C concentrations observed in Japanese children compared with American and Australian children were proposed to help explain the low CHD mortality in Japan relative to other developed countries [11].

In Spain, a country characterized by a low CHD mortality rate compared to that in other developed countries [12], we have described that the mean concentration of plasma HDL-C levels in children remained stable and high over time [13]. Comparing plasma cholesterol levels in prepuberal Spanish children across successive surveys during the last decades, we observed that while total cholesterol increased among 6–8-year-old children, mean HDL-C remained high (59.0 mg/dL) without significant variation over time [13], which may help to explain the low CHD mortality rate in Spain.

These consistently high HDL-C concentrations in Spanish children support the existence of a distinctive combination of genetic, environmental and/or population characteristics contributing to them and justify our interest in analyzing these factors.

HDL-C levels are influenced by a complex interaction of behavioral, environmental, metabolic and genetic factors [14]. Two possible initial interpretations for the high and stable levels of HDL-C in our children could be that a diet increasingly rich in fats in our country has reached its maximum effect on HDL-C, although low-density lipoprotein cholesterol (LDL-C) continues to rise [13], or that the stability of HDL-C has a genetic basis, although the underlying causes are likely to be multifactorial.

In this context, our group has worked over the last decades on the Four Provinces Study (4P), an epidemiological, cross-sectional study that aims to analyze cardiovascular risk factors in Spain, initially focused on comparing lipid levels in children among regions in Spain with different CHD mortality rates: high in Cádiz and Murcia and low in Madrid and Orense [15]. Children were selected from clusters of schools by random sampling, stratified by sex and type of school (public vs. private). Fieldwork was conducted between 1998 and 2000, in the same season in each province [15]. The prepubertal cohort studied in the 4P Study, comprising 1275 children aged 6–8 years (643 boys and 632 girls), is a good model to analyze factors contributing to HDL-C concentration, as the prepubertal period is a concrete metabolic window without the influence of confounding factors known to affect HDL-C, such as smoking, alcohol intake or sex steroids, which operate in other age groups. The sample size varied slightly among analyses depending on the data available for each variable. Anthropometry was measured by trained staff, and body mass index (BMI) was calculated. Obesity was defined according to the age- and sex-specific BMI cut-off points proposed by Cole et al. [16] for children. Venous blood (12-h fasting) was collected from each participant to obtain plasma, serum and DNA. Particularly, fasting HDL-C was determined in a certified laboratory. The study protocol was approved by the Clinical Research Ethics Committee of the Fundación Jiménez Díaz (Madrid), complied with the Declaration of Helsinki, and written informed consent was obtained from the parents.

In this review, we summarize and critically discuss the evidence regarding factors affecting HDL-C levels in prepubertal children, accumulated over more than two decades of research in the Four Provinces Study, to advance the knowledge of factors related to HDL-C concentrations in children.

## 2. Metabolic Determinants

### 2.1. Obesity and HDL-Cholesterol

Dyslipidemia is one of the most frequent obesity-related comorbidities in childhood. Classical epidemiological studies of cardiovascular risk factors in children and young adults, such as the Bogalusa Heart Study [17], the Taipei Children Heart Study [18], and the CATCH study [19] have described that increased adiposity in children is accompanied by insulin resistance and a more adverse lipid profile, establishing the metabolic context in which lower HDL-C may emerge.

A systematic review and meta-analysis of cardiovascular risk factors in children also reported associations between higher BMI and an unfavorable lipid profile, including lower HDL-C [20]. In our cohort of prepubertal children, mean HDL-C concentrations were lower in children with obesity than in their normal-weight counterparts (mg/dL, mean ± SD: 52.5 ± 10.7 vs 60.1 ± 13.0, *p* < 0.001 in boys; 54.8 ± 15.7 vs 58.5 ± 12.8, *p* = 0.05 in girls) [21]. However, when performing Spearman correlation analysis, BMI was not significantly correlated with HDL-C [21].

Therefore, HDL-C may not depend exclusively on the amount of body fat mass, here assessed by BMI, but also on metabolic alterations or aspects linked to adipose tissue, such as insulin sensitivity and adipokines, which led us to investigate the role of insulin, leptin, and other adipokines in the association between obesity and HDL-C.

### 2.2. Insulin Sensitivity and HDL-Cholesterol

In our cohort, insulin concentrations and the homeostasis model assessment of insulin resistance (HOMA-IR) were positively associated with BMI, as expected. In a multivariable regression analysis, neither insulin nor HOMA-IR was significantly associated with plasma HDL-C after adjusting for BMI [22]. Further exploring this relationship, when comparing HDL-C concentrations in boys and girls with obesity with the concentrations in their normal-weight counterparts, considering insulin as a covariable, we observed that HDL-C concentrations in boys with obesity remained significantly lower than the concentrations in normal-weight boys (Table 1). In contrast, in girls, including insulin as a covariable, significant differences in HDL-C levels between weight category groups disappeared (Table 1). Similar results were observed when HOMA was included in the regression analysis.

Our results suggest that at this age, the relationship between obesity, insulin resistance, and HDL-C differs by sex. In boys, the lower HDL-C concentrations associated with obesity appear to be independent of insulin and HOMA, whereas in girls, insulin and HOMA-IR were associated with HDL-C concentrations and may contribute to the observed differences in HDL-C. These sex-related associations are particularly striking given that insulin and HOMA levels did not differ significantly between boys and girls with obesity, although normal-weight girls had significantly higher insulin concentrations than normal-weight boys (Table 2).

Insulin may affect HDL metabolism through several interconnected pathways. These include increased hepatic production of very-low-density lipoproteins (VLDL), cholesteryl ester transfer protein (CETP)-mediated exchange of cholesteryl esters and triglycerides between HDL and ApoB-containing lipoproteins and increased hepatic lipase activity [23]. Such processes can promote triglyceride enrichment and subsequent remodeling and accelerated catabolism of HDL particles [24]. Whether these mechanisms account for the associations observed in our prepubertal cohort cannot be determined from our cross-sectional analyses.

In summary, in prepubertal children, the influence of insulin sensitivity on the association between obesity and HDL-C seems to differ by sex: in boys, lower HDL-C associated with obesity appears to be largely independent of insulin resistance, but not in girls.

### 2.3. Leptin and HDL-Cholesterol

Among the most relevant results derived from our metabolic analyses of HDL-C in prepubertal children were those concerning the relationship between circulating leptin and lipid concentrations, studied in our population-based sample [25]. To our knowledge, this is one of the few population-based studies analyzing the relationship between leptin and lipid profiles in a cohort of prepubertal Caucasian children. Our main finding was that in children aged 6–8 years, higher leptin concentrations were associated not only with higher insulin concentrations but also with lower HDL-C, an association that remained significant in boys after adjustment for BMI.

Regarding these sex-related differences, when leptin was analyzed as a continuous variable, its inverse association with HDL-C remained significant after adjustment for BMI or insulin in boys, but not in girls (Table 3). Thus, in boys, circulating leptin appeared to be associated with HDL-C independently of both adiposity, estimated by BMI, and insulin concentrations.

However, when analyzing HDL-C according to leptin tertiles, we observed a different feature of this association. In both sexes, children in the highest leptin tertile had lower HDL-C concentrations, whereas HDL-C did not differ substantially between the low and intermediate leptin tertiles [25], suggesting that the relationship between leptin and HDL-C may not be entirely linear. Interestingly, after adjustment for BMI or insulin, the differences across leptin tertiles disappeared in boys but remained highly significant in girls. Although these findings may initially appear contradictory, continuous and categorical analyses may capture different aspects of the leptin–HDL-C relationship. In boys, the association between leptin and HDL-C appears to be relatively continuous but closely linked to increasing adiposity. In contrast, in girls, the persistence of lower HDL-C specifically among those with the highest leptin concentrations after adjustment for BMI or insulin suggests a possible threshold effect: very high leptin concentrations may convey metabolic information that is not adequately captured by BMI alone. This non-linear pattern could also explain why an independent association was not apparent when leptin was modelled as a continuous variable.

Consistent with the close interrelationships among these metabolic variables, the weak inverse association between insulin and HDL-C observed in both sexes disappeared after adjustment for leptin, suggesting that leptin may account for part of the relationship between insulin and HDL-C (Table 3).

Previous studies in children of different ages and ethnicities have also reported a negative association between leptin and HDL-C levels [26,27,28]. In experimental studies, low-dose leptin treatment markedly lowered HDL-C in both leptin-deficient (*ob/ob*) and wild-type mice without significant changes in body weight and restored hepatic apoA-I mRNA to normal levels in *ob/ob* mice [29].

The association between leptin and HDL-C may reflect the complex interplay between adipose tissue function and systemic lipid metabolism. Although the design of our study does not allow us to elucidate the underlying mechanisms of this relationship, several potential mechanisms may be proposed. Among the mechanisms that could link leptin to HDL-C, leptin may modify the expression or activity of proteins involved in lipoprotein metabolism, promoting changes in the size and composition of HDL or modulating cholesterol transport from peripheral tissues to the liver. In addition, leptin resistance is often associated with increased VLDL and hypertriglyceridemia. Through CETP, HDL exchanges cholesteryl esters for triglycerides with VLDL, and triglyceride-enriched HDL particles are more readily hydrolyzed by hepatic lipase and cleared from plasma, which lowers HDL-C [23,30,31]. These mechanisms should, however, be regarded as plausible explanations rather than causal relationships established by our findings. Further investigation is needed to clarify these mechanisms and the marked sex differences observed.

In summary, our findings suggest that leptin should not be regarded simply as a marker of adipose mass in relation to HDL-C. Rather, the relationship appears to be sex-dependent and potentially non-linear. Particularly in girls, high circulating leptin may identify metabolic alterations beyond those reflected by BMI, although these observational data cannot establish whether this represents leptin resistance or a direct effect of leptin on HDL metabolism. Thus, adipose-tissue endocrine activity may provide additional information beyond anthropometric measures of adiposity in understanding interindividual variability in HDL-C.

## 3. Genetic Determinants

As we discussed previously, one possibility to explain the stability of HDL-C levels in our children and the differences between countries could be that these differences are genetically based. HDL-C is now recognized as a highly polygenic trait, with genome-wide association studies identifying several hundred loci, each with a small individual effect [32]. We have investigated several potential genetic determinants of HDL-C, including single-nucleotide variants (SNVs) in genes directly involved in HDL metabolism and in genes encoding molecules that indirectly affect HDL levels.

### 3.1. Direct Genetic Determinants of HDL-Cholesterol

In our paper entitled “Genetic determinants of plasma HDL-C levels in prepubertal children” [33], which included 1269 healthy children aged 6–8 years, we examined SNVs of genes directly involved in HDL metabolism: *APOA*1 MspI−75A/G (rs670), *APOA*1 MspI +83 C/T (rs5069), *APOA2* MspI C/T (+528 bp 3′; M1/M2), *CETP* TaqIB (rs708272), *LIPC* C-514T (rs1800588), *ABCA1 R219K (*rs2230806), *ABCA1* V771M (rs2066718), *PON1* 192Q/R (rs662), and *PON1* 55L/M (rs854560).

The main finding of our analysis was that the *CETP* TaqIB polymorphism was the principal genetic predictor of HDL-C. Children carrying the B2 allele had higher HDL-C, although the genotype pattern differed somewhat between boys and girls. TaqIB explained approximately 4.5% of HDL-C variability in girls and 1.8% in boys [33]. None of the other studied variants made a comparable independent contribution.

Kondo et al. [34] were among the first to describe a relationship between HDL-C plasma levels and polymorphisms at the locus for human *CETP*. Our finding is consistent with a large meta-analysis in adults, in which the B2 allele was associated with lower CETP mass and activity and higher HDL-C [35]. Interestingly, the sex pattern we observed (a stronger effect in girls) contrasts with that reported in some other populations, where the *CETP* TaqIB effect on HDL-C has been described as stronger in men [36], suggesting that sex-specific modulation of this association may vary depending on age and across populations.

On the other hand, other genes with important implications for lipid metabolism, such as apolipoprotein E (*APOE*), do not appear to be related to HDL-C in our children. Apolipoprotein E is a key regulator of plasma lipid levels, which affects the hepatic binding, uptake, and catabolism of several classes of lipoproteins [37]. Previous studies have documented the impact of the common *APOE* polymorphism (ε2/ε3/ε4) on the normal variation in plasma total cholesterol (TC) and LDL-C levels in children [38,39], although its effect on HDL-C is notably weaker and less consistent than on LDL-C and total cholesterol, even in large meta-analyses [40]. In the Four Provinces Study, we found a strong influence of *APOE* genotype on the metabolism of the Apo B-containing lipoproteins but not on HDL-C levels [41,42,43].

Recently, we also examined two lipoprotein lipase (*LPL*) variants, rs258 and rs326 [44]. Because LPL-mediated lipolysis of triglyceride-rich lipoproteins provides surface components for HDL formation, *LPL* variation could link triglyceride metabolism, adipose tissue signaling, and HDL-C. However, although these variants were associated with other components of the lipid profile and with leptin concentrations, no independent association with HDL-C was observed.

Overall, these studies indicate that, among common variants in genes directly involved in HDL metabolism, *CETP* TaqIB emerged as the strongest predictor of HDL-C in prepubertal children. Nevertheless, its modest explanatory contribution, consistent with the polygenic architecture of HDL-C described above, indicates that most interindividual HDL-C variability remains attributable to other genetic, metabolic, and environmental factors or to an interaction of these factors.

### 3.2. Indirect Genetic Determinants of HDL-Cholesterol

Based on the observed influence of leptin on HDL-C, we analyzed SNVs in genes of adipokines and other molecules involved in energy homeostasis or metabolic signaling that may be indirectly associated with HDL-C, such as leptin (*LEP*), adiponectin (*ADIPOQ*), perilipins 1 and 2 (*PLIN1*, *PLIN2*), and delta-like non-canonical Notch ligand 1 (*DLK1*).

#### 3.2.1. Leptin Gene Variants and HDL-C

In our study, we investigated the potential relationship of SNVs 19G>A (rs2167270) and −2548G>A (rs7799039) of the *LEP* gene with lipid variables. We found that both *LEP* SNVs were associated with leptin levels after adjusting for sex. When analyzing their association with lipid levels, we observed that the −2548G>A SNV was also associated with HDL-C levels after adjusting for sex and that the association remained statistically significant after adjusting for circulating leptin levels; no association was found with the remaining lipid variables (LDL-C, triglycerides, total cholesterol) [45]. This finding suggests that the influence of genetic variants in the leptin gene on HDL metabolism may not be mediated exclusively through systemic leptin concentrations and may influence lipid metabolism through alternative mechanisms. Because −2548G>A lies in the promoter region, it could modify local (autocrine/paracrine) leptin expression in adipose tissue without detectable changes in circulating leptin, or be in linkage disequilibrium with other functional variants. Leptin signaling also regulates hepatic HDL handling, including the expression of the HDL receptor scavenger receptor class B type I (SR-BI), which mediates selective HDL cholesterol uptake and thereby influences HDL clearance and ApoA-I turnover [46,47]. This result is relevant because it implies that the association between *LEP* variants and HDL-C may involve direct or indirect effects on lipid-processing pathways rather than being explained solely by blood leptin concentrations.

#### 3.2.2. Perilipin Variants and Leptin-Modulated Effects

A role for leptin in HDL-C-related pathways also emerged when we examined the association of SNVs at the perilipin (*PLIN*) locus with lipid levels and assessed whether plasma leptin mediated or modified these associations. Perilipins are proteins that coat intracellular lipid droplets in adipocytes and other cell types, including hepatocytes, in which PLIN2 is the predominant isoform, and play a central role in lipid storage and mobilization, regulating access of lipases to stored triglycerides [48,49,50]. In our study, we analyzed the SNVs rs894160, rs1052700, and rs2304795 in *PLIN1*, and rs35568725 in *PLIN2.* We found a significant association of the SNV *PLIN2* rs35568725 with HDL-C in boys but not in girls. In boys, G (AG + GG) carriers showed significantly lower HDL-C levels than AA carriers, without showing significant differences in girls [51].

Moreover, when the analysis was stratified by leptin concentrations, we observed that the association of *PLIN1* SNVs with HDL-C emerged depending on leptin concentrations; specifically, an association was found between *PLIN1* rs1052700 and HDL-C in girls with high leptin levels. This association was not observed in boys.

Taken together, the sex-specific association of *PLIN2* rs35568725 with HDL-C and the fact that the associations of *PLIN1/PLIN2* variants with HDL-C appear to be modulated by leptin levels are consistent with a genotype × sex × leptin interaction in relation to HDL-C levels. Leptin concentrations may modulate the effect of *PLIN* variants, and sex-based differences in leptin levels could help explain why the same genetic variant shows different associations in boys and girls. Our data allow us to speculate that sex differences in plasma leptin concentrations throughout life, affecting the relationship of the *PLIN* SNVs with HDL-C, may contribute to explaining the differences in HDL-C concentrations between sexes across the life course.

Furthermore, the association between *PLIN* SNVs and HDL-C suggests that inherited variation in genes controlling adipocyte lipid storage and mobilization may contribute to differences in circulating HDL cholesterol. The SNVs could alter *PLIN* expression or function—or simply be linked to another functional variant—and thereby affect fatty acid release, triglyceride metabolism, and ultimately HDL metabolism.

In summary, the *PLIN* SNV–HDL-C association suggests that genetic variation in lipid droplet regulation may influence systemic lipid metabolism. Further studies analyzing these aspects in other age groups should be performed, as well as functional validation.

#### 3.2.3. DLK1 SNVs and HDL-Cholesterol

Our findings regarding the association of perilipin genetic variants and HDL-C support an integrated pathway linking adipocyte lipid storage, adipose tissue signaling, and circulating lipoproteins, supporting a connection between adipose-tissue lipid handling and HDL metabolism. In this sense, we also analyzed the association between genetic variants of *DLK1* and circulating HDL-C levels. DLK1 is encoded by a paternally expressed imprinted gene at the *DLK1–MEG3* locus (14q32) and is involved in adipocyte differentiation [52,53], acting as a modulator of adipogenesis. Five *DLK1* SNVs (rs1802710, rs876374, rs7155375, rs57098752, and rs7149242) were analyzed, finding that the SNV rs57098752 was significantly associated with circulating HDL-C concentrations, independently of BMI, in boys [54]. Notably, rs1802710 had previously been associated with childhood and adolescent obesity through a parent-of-origin (polar overdominance) effect [55], supporting the relevance of this locus for pediatric metabolic traits.

Our study links a gene involved in adipogenesis and developmental regulation with plasma lipid traits in prepubertal children. Replication in independent cohorts and functional studies are needed to confirm this association and clarify its mechanism.

The genetic associations discussed in this review should be interpreted with caution. In the relevant original studies, correction for multiple testing was not performed, which may increase the risk of false-positive findings and limits the strength of the conclusions that can be drawn from these associations. Independent replication in adequately powered and, where possible, independent populations is therefore necessary to assess the robustness and generalizability of these findings.

## 4. Diet and HDL-Cholesterol

Diet is another potential determinant of HDL-C concentrations in school-age children. Traditionally, differences in HDL-C levels between countries have been attributed to dietary factors, suggesting that the influence of diet on HDL-C is more evident at the interpopulation level than within populations [56,57]. It is well known that different dietary components affect HDL-C levels [58]. When they are isoenergetically replacing carbohydrates, all classes of dietary fatty acids raise HDL-C, with the greatest effect for saturated, intermediate for monounsaturated, and smallest for polyunsaturated fatty acids; conversely, high-carbohydrate diets and excess energy intake leading to weight gain lower HDL-C [59,60]. However, observational data on nutrient intake and HDL-C remain inconsistent across studies [61,62].

From a nutritional epidemiology perspective, olive oil plays a central role in the Spanish diet. Studies by Prof. de Oya showed that diets enriched in monounsaturated fatty acids, besides lowering TC and LDL-C, maintain or raise HDL-C [63]. However, HDL-C levels in our cohort of prepubertal children could not be attributed to monounsaturated fat intake. Our data suggest that the high HDL-C levels in our children may be partly related to the high levels of total fat and saturated fat they consume, which may mask the potential positive effect of monounsaturated fat. Dietary assessment in our cohort through a food frequency questionnaire (FFQ) [64] adapted to children showed that total fat intake and the percentage of energy from saturated fat exceeded recommendations [65]. Our study characterized saturated fat consumption and its relationships with anthropometric, dietary, nutritional, and lipid profiles, finding that, in addition to its strong association with total cholesterol and LDL-C, saturated fat intake was associated with HDL-C and Apo-AI, and particularly with the ratio LDL-C/HDL-C [66]. In adults and young children, modification of dietary fat intake leads to parallel changes in HDL-C and non-HDL-C [56,67].

Another possible explanation for these HDL-C levels could be low carbohydrate consumption. Data from Knuiman et al. [56] showed that 7–9-year-old boys from countries with a higher percentage of energy derived from carbohydrates had lower HDL-C levels. In our population we found a significant, but small, negative correlation between simple carbohydrate intake and HDL-C only in boys (unpublished data).

Alcohol intake raises HDL-C levels in adults; the absence of alcohol consumption in our prepubertal cohort allowed us to evaluate dietary factors affecting HDL-C without this confounder.

When evaluating dietary variety, greater dietary variety was associated with better biochemical nutritional status, being somehow related to HDL-C concentrations [68]. Children in the upper tertile of the diet variety index (DVI) had slightly higher HDL-C and lower triglyceride concentrations than those in the other tertiles, although these data do not establish a direct relationship between dietary variety and HDL-C.

In summary, diet appears to play only a modest role in explaining HDL-C concentrations in prepubertal children in our country. However, we should mention as an important limitation to this conclusion the fact that dietary information was obtained using an FFQ rather than with more precise methods such as weighed food records or repeated 24-h dietary recalls. Thus, further controlled studies should be performed to evaluate the relationship of fat and carbohydrate intake with HDL-C levels in our children.

## 5. Dietary Modulation and Gene–Nutrient Interactions

Beyond the modest direct association between dietary intake and HDL-C, we have observed that the genetic effect on HDL-C of different variants in genes directly and indirectly related to HDL-C levels is not necessarily fixed but rather may depend on the nutritional context.

A notable example is the interaction between the hepatic lipase gene (*LIPC*) and fat intake. When analyzing the *LIPC* −514C>T (rs1800588) polymorphism in relation to lipid levels in healthy children aged 6–8 years, we observed that carriers of the minor *LIPC* C-514T allele had higher ApoA-I concentrations, regardless of sex. More importantly, the lipid effect of the genotype was modified by dietary fat intake: in boys, carriers of the minor *LIPC* C-514T allele had significantly higher HDL-C among those in the highest tertiles of total, saturated, monounsaturated, or polyunsaturated fat intake [69].

Our data show that dietary fat intake modifies the effect of the *LIPC* C-514T polymorphism on plasma HDL-C and ApoA-I levels in prepubescent children, being associated with higher levels of HDL-C only when fat intake is high. This gene–nutrient interaction could help to explain interindividual variation in the plasma lipid response to fat intake. Interestingly, its direction differs from that described in adults from the Framingham Study, in whom the T allele was associated with higher HDL-C only when fat intake was below 30% of energy, whereas TT homozygotes had the lowest HDL-C when fat intake was ≥30% of energy [70]. Differences in age, the very high fat intake of our population, and the absence of insulin resistance and alcohol intake in children may account for this discrepancy, which further suggests that genetic influences on childhood HDL metabolism depend on the nutritional context.

In summary, these data suggest that a lipid-related genetic association could depend on nutritional exposure rather than operate as a fixed genotype effect.

## 6. Sex-Dependent Associations Before Puberty

One of the most consistent findings across our studies is that sex acts as an effect modifier of HDL-related traits already in prepubertal children. Sex-dependent associations were observed for obesity and insulin resistance, leptin, *CETP*, perilipin variants and *DLK1*, among other metabolic and genetic determinants. Thus, sex should not be considered merely a covariate in childhood HDL metabolism, but rather a biological factor that modifies the relationships between adiposity, metabolic signaling, genetic background and HDL-C.

The mechanisms underlying these differences remain unclear. Adipose-tissue signaling may contribute, as suggested by the marked sex differences observed in the associations between leptin and HDL-C and in leptin-dependent genetic effects. However, subtle differences in endocrine maturation may also be relevant even before overt puberty. In this context, we found no association between DHEAS and HDL-C in normal-weight children, whereas among children with overweight, boys in the highest DHEAS tertile had significantly lower HDL-C concentrations; this association was not observed in girls [71]. This finding suggests that adrenal maturation may interact with sex and metabolic/adiposity context in the regulation of HDL-C, rather than exerting a uniform effect on HDL-C during childhood. Our data regarding DHEAS suggest that the absence of overt pubertal gonadal maturation does not mean absence of developmental endocrine variation.

## 7. Methodological Limitations

Several methodological considerations should be considered when interpreting the findings summarized in this review. At the same time, these limitations identify important areas for future research into the determinants of HDL-C during childhood.

A limitation common to most epidemiological studies is the lack of information on body composition and fat distribution. Adiposity was assessed using BMI, which is a practical measure in population studies but does not distinguish fat mass from lean mass or provide information on the amount and distribution of adipose tissue. This distinction may be particularly relevant to our findings. Although children with obesity generally had lower HDL-C concentrations, BMI itself was not consistently associated with HDL-C, whereas variables related to adipose-tissue function, particularly leptin, showed stronger and sex-dependent associations. Future studies incorporating direct measures of body composition could help to clarify these aspects.

The lack of physical activity information represents another important limitation. Physical activity may influence HDL metabolism independently of adiposity and could also modify the relationships between HDL-C, insulin sensitivity, and adipokines. The absence of measurements of physical activity limits our ability to distinguish the contributions of diet, adiposity, metabolic status and habitual activity to interindividual differences in HDL-C.

The assessment of dietary intake also has limitations. Dietary information in the Four Provinces Study was obtained using a food-frequency questionnaire. Although this approach is suitable for characterizing habitual dietary patterns in large epidemiological studies, measurement error and imprecision in the estimation of individual nutrient intake are unavoidable. This limitation is particularly important when evaluating relatively modest associations or gene–nutrient interactions. Therefore, the apparently limited contribution of diet to HDL-C variability should be interpreted cautiously. Studies combining repeated dietary assessments with more detailed methods, such as 24-h dietary recalls or food records, would provide a more precise evaluation of the relationships between fat quality, carbohydrate intake, and HDL metabolism.

A further limitation derives from the observational nature of most of the analyses summarized here. Associations involving leptin, insulin, adiposity, diet, or genetic variants cannot establish causal relationships or determine the direction of the biological pathways involved. This is particularly relevant for leptin. Our findings support a close relationship between leptin and HDL-C, but they cannot determine whether leptin directly influences HDL metabolism, whether circulating leptin is predominantly a marker of adipose tissue dysfunction, or whether both variables reflect other underlying metabolic processes. Further mechanistic and longitudinal studies are needed to clarify the role of leptin in HDL metabolism and to establish the nature and direction of the relationship between adipose tissue function and HDL-C levels.

Also, sex-related differences in the associations between leptin, genetic variants, and HDL-C should be interpreted cautiously. Although some associations were observed in one sex but not the other, differences in statistical significance between boys and girls do not necessarily indicate a statistically significant difference between sexes. Formal interaction analyses were not consistently available across all the original studies reviewed, limiting the extent to which sex-specific effects can be established. Further studies with adequate statistical power and formal tests of interaction are needed to determine whether the associations between adipose tissue-related factors, genetic variants, and HDL-C genuinely differ by sex.

Finally, we should acknowledge that HDL-C levels in our study may be attributable to genetic, cultural and dietary characteristics specific to the Spanish population, which may limit the direct generalizability of our findings to other populations.

## 8. Future Directions

The findings derived from the Four Provinces Study raise several questions that remain unresolved and may help define future research on HDL metabolism in childhood.

*What may explain the early emergence of sex differences in HDL regulation?* One of the most consistent observations across our studies is that sex modifies several associations with HDL-C, including those involving obesity and insulin resistance, leptin, *CETP*, *PLIN* and *DLK1* variants. Importantly, these differences are already detectable before overt puberty. Therefore, the major question is therefore which biological mechanisms generate this sexual dimorphism at such an early age. Longitudinal studies incorporating detailed measures of pubertal development, adrenal and gonadal hormones, body composition, and adipose tissue function will be required to determine whether these differences precede hormonal maturation, reflect subtle differences in maturation already present during the prepubertal period, or result from other sex-dependent metabolic or genetic mechanisms.

*Is adipose-tissue function more important than the amount of adiposity in determining HDL-C?* BMI alone does not adequately explain the variation in HDL-C observed in our population, whereas leptin and other metabolic characteristics associated with adipose tissue appear to provide additional information. This raises the possibility that the metabolic and endocrine state of adipose tissue may be more relevant to HDL metabolism than body size per se. Future studies combining direct measurements of total and regional adiposity with adipokines, insulin sensitivity and markers of adipose tissue dysfunction could establish whether children with similar degrees of adiposity have different HDL phenotypes depending on the functional state of their adipose tissue.

*What is the biological basis of the relationship between leptin and HDL metabolism?* The association between leptin and HDL-C is among the most intriguing findings emerging from our studies. However, it remains unclear whether leptin has a direct role in HDL metabolism or predominantly acts as a marker of adiposity, leptin resistance or broader metabolic dysfunction. The apparent non-linearity and sex dependence of this relationship further suggest that different mechanisms may operate according to the metabolic context. Functional studies are needed to investigate whether leptin signaling influences pathways involved in HDL production, remodeling or clearance and whether these effects differ between boys and girls.

## 9. Conclusions

Studies conducted in this cohort indicate that plasma HDL-C concentrations in prepubertal children result from a complex interplay of genetic, metabolic and environmental factors. Common variants in classical HDL-metabolism genes explain only a small proportion of interindividual HDL-C variability. *CETP* TaqIB emerged as the strongest candidate-gene determinant identified, but its explanatory contribution was modest, indicating that individual genetic variants alone cannot account for the high HDL-C concentrations or their variability in this population.

Importantly, genetic effects appear to be context-dependent rather than fixed. Dietary fat, adiposity, leptin concentrations and sex can modify genetic associations with HDL-C, highlighting the relevance of gene–environment and gene–metabolic interactions. Diet itself appears to make only a modest contribution to HDL-C variability, with associations mainly involving saturated fat and carbohydrate intake.

Among the factors examined, metabolic status, and particularly adipose-tissue signaling, emerged as especially relevant. Our findings suggest that adipose-tissue dysfunction may provide information beyond body weight alone: leptin, insulin sensitivity and related metabolic alterations represent plausible links between adiposity and HDL metabolism. The recurrent involvement of leptin, both directly and as a modifier of genetic associations, further supports the importance of the adipose tissue–HDL axis in childhood.

A second major finding was the consistent role of sex as a biological effect modifier. Sex-dependent associations involving adiposity, insulin resistance, leptin and several genetic determinants are already detectable before clinical puberty. These observations suggest that sex-specific regulation of HDL metabolism begins early in life and may arise from interactions among genetic background, adipose-tissue signaling, metabolic status and early endocrine maturation.

Overall, our findings suggest that the variability of HDL-C in prepubertal children is better understood through the metabolic and biological context in which genetic and environmental factors operate than through their individual effects alone. In this framework, adipose-tissue signaling, particularly leptin, and sex-dependent regulation emerge as two prominent features that may help explain part of the substantial interindividual variability in childhood HDL-C.

## Figures and Tables

**Table 1:** Insulin-adjusted HDL-C concentrations (mg/dL) in normal-weight children and children with obesity, by sex.

		**Boys**	
	*n*	HDL-C	*p*-value
Normal weight	378	59.7 ± 0.7	<0.001
Obesity	36	51.2 ± 2.2	
		**Girls**	
	*n*	HDL-C	*p*-value
Normal weight	378	58.3 ± 0.7	0.396
Obesity	43	56.5 ± 2.0	

ANCOVA. Estimated marginal means ± SE.

**Table 2:** Insulin concentrations and HOMA-IR by sex and weight category (mean ± SD).

**Normal Weight**		
	Insulin (µU/mL) (*n*)	HOMA-IR (*n*)
Boys	3.1 ± 2.0 (381)	0.7 ± 0.5 (378)
Girls	3.4 ± 2.4 (379)	0.8 ± 0.5 (377)
*p*-value	0.022	0.175
**Obesity**		
	Insulin (µU/mL) (*n*)	HOMA-IR (*n*)
Boys	5.4 ± 2.6 (36)	1.3 ± 0.7 (36)
Girls	5.7 ± 3.9 (43)	1.3 ± 0.8 (43)
*p*-value	0.596	0.976

*p*-value: Student’s T-test. Obesity was defined according to Cole et al. [16].

**Table 3:** Spearman correlation coefficients (unadjusted and partial) between HDL-C and BMI, leptin and insulin, by sex.

**Boys**				
HDL-C (mg/dL)				
	Non-adjusted	Adjusted for BMI	Adjusted for leptin	Adjusted for insulin
BMI (kg/m^2^)	−0.043	-	0.013	−0.088
Leptin (ng/mL)	−0.143 ***	−0.136 **	-	−0.160 ***
Insulin (µU/mL)	−0.090 *	−0.021	0.014	-
**Girls**				
HDL-C (mg/dL)				
	Non-adjusted	Adjusted for BMI	Adjusted for leptin	Adjusted for insulin
BMI (kg/m^2^)	−0.073	-	−0.028	−0.055
Leptin (ng/mL)	−0.119 **	−0.052	-	−0.060
Insulin (µU/mL)	−0.097 *	−0.082	−0.073	-

*p* < 0.05 *; *p* < 0.01 **; *p* < 0.001 ***.

## Data Availability

No new data were created or analyzed in this study. Data sharing is not applicable to this article.

## Participants of the Four Provinces Study

Manuel de Oya; Carmen Garcés; Fernando Rodríguez-Artalejo; Ángel Gil; Miguel A. Lasunción; Henar Ortega-Senovilla; Rafael Rubio; Mercedes Benavente; José L. del Barrio; José Mª Martín Moreno; Lydia Gorgojo; Miguel A. Royo-Bordonada; Ovidio Fernández; Alipio Mangas; Alfonso Macías; Jacinto Fernández Pardo; Alberto Torres-Cantero; Mª José Bayonas; Enrique Viturro; Beatriz Cano; Laura López-Simón; Iria de Oya; Pía Riestra; Stefanie Schoppen; Alicia García-Anguita; Lorena Ortega; Leandro Soriano-Guillén; Teresa Gavela-Pérez; Pilar Navarro; Asha Jois; Olaya de Dios; Claudia Vales-Villamarín; Jairo Lumpuy-Castillo; Olga Pomares; Jorge Laborda.

## Abbreviations

The following abbreviations are used in this manuscript:

ABCA1	ATP-binding cassette subfamily A member 1
ADIPOQ	Adiponectin
ApoA-I	Apolipoprotein A-I
ApoB	Apolipoprotein B
ApoE	Apolipoprotein E
BMI	Body mass index
CETP	Cholesteryl ester transfer protein
CHD	Coronary heart disease
DHEAS	Dehydroepiandrosterone sulfate
DLK1	Delta-like non-canonical Notch ligand 1
HDL-C	High-density lipoprotein cholesterol
HOMA-IR	Model assessment of insulin resistance
LDL-C	Low-density lipoprotein cholesterol
LEP	Leptin
LIPC	Hepatic lipase
LPL	Lipoprotein lipase
PON1	Paraoxonase 1
SNV	Single nucleotide variant
TC	Total cholesterol
VLDL	Very low-density lipoprotein
